# Cysteine cathepsin activity suppresses osteoclastogenesis of myeloid-derived suppressor cells in breast cancer

**DOI:** 10.18632/oncotarget.4714

**Published:** 2015-07-17

**Authors:** Laura E. Edgington-Mitchell, Jai Rautela, Hendrika M. Duivenvoorden, Krishnath M. Jayatilleke, Wouter A. van der Linden, Martijn Verdoes, Matthew Bogyo, Belinda S. Parker

**Affiliations:** ^1^ Department of Biochemistry and Genetics, La Trobe Institute for Molecular Science, La Trobe University, Melbourne, Australia; ^2^ Sir Peter MacCallum Department of Oncology, The University of Melbourne, Parkville, Australia; ^3^ Department of Pathology, Stanford University School of Medicine, California, USA; ^4^ Department of Tumour Immunology, Radboud Institute for Molecular Life Sciences, Nijmegen, The Netherlands; ^5^ Drug Discovery Biology, Monash Institute of Pharmaceutical Sciences, Melbourne, Australia

**Keywords:** cysteine protease, cathepsin, myeloid-derived suppressor cell, breast cancer metastasis, osteoclast

## Abstract

Cysteine cathepsin proteases contribute to many normal cellular functions, and their aberrant activity within various cell types can contribute to many diseases, including breast cancer. It is now well accepted that cathepsin proteases have numerous cell-specific functions within the tumor microenvironment that function to promote tumor growth and invasion, such that they may be valid targets for anti-metastatic therapeutic approaches. Using activity-based probes, we have examined the activity and expression of cysteine cathepsins in a mouse model of breast cancer metastasis to bone. In mice bearing highly metastatic tumors, we detected abundant cysteine cathepsin expression and activity in myeloid-derived suppressor cells (MDSCs). These immature immune cells have known metastasis-promoting roles, including immunosuppression and osteoclastogenesis, and we assessed the contribution of cysteine cathepsins to these functions. Blocking cysteine cathepsin activity with multiple small-molecule inhibitors resulted in enhanced differentiation of multinucleated osteoclasts. This highlights a potential role for cysteine cathepsin activity in suppressing the fusion of osteoclast precursor cells. In support of this hypothesis, we found that expression and activity of key cysteine cathepsins were downregulated during MDSC-osteoclast differentiation. Another cysteine protease, legumain, also inhibits osteoclastogenesis, in part through modulation of cathepsin L activity. Together, these data suggest that cysteine protease inhibition is associated with enhanced osteoclastogenesis, a process that has been implicated in bone metastasis.

## INTRODUCTION

Breast cancer metastasis to lung and bone poses a serious threat to life. Cysteine cathepsins comprise a family of proteases that have been widely implicated in primary breast tumor invasion and progression [1–5]. We have recently demonstrated a critical role for tumor-derived cathepsin B using the syngeneic 4T1.2 model of murine breast cancer metastasis [6]. Tumor cell knockdown of cathepsin B significantly abrogated metastasis to lung and bone. Likewise, enforced tumor cell expression of an endogenous cysteine cathepsin inhibitor, Stefin A, had similar effects [7], as did systemic treatment with a small-molecule cathepsin B inhibitor, CA074 [6].

In addition to their roles within tumor cells, cysteine cathepsins have been shown to have disparate functions in stromal cells within the tumor microenvironment [4, 8–10]. Blocking protease activity may therefore have varied effects depending on the target cell, timing of administration, and the specificity of any inhibitor for a particular target protease. For this reason, protease inhibitors have largely failed as therapeutic agents in the clinic [11, 12]. To improve their chance of success, a more detailed understanding of the cell-specific localization, timing and function of protease activation is absolutely essential. The 4T1 series of murine breast cancer cells is ideal for studying cell-specific roles of proteases since it utilizes syngeneic immunocompetent mice [13–15]. This allows for accurate cross-talk between tumor and host cells, including immune cells. This isogenic series comprises cells that are highly metastatic to lung and bone (4T1.2) and those that are non-metastatic (67NR) [14], allowing assessment of the contribution of tumor and stromal cysteine cathepsins to metastatic progression.

Myeloid-derived suppressor cells (MDSCs) are one type of immature immune cell that dramatically expands during 4T1 metastasis [16–19], as well as in breast cancer patients [20, 21]. The canonical function of these cells is to suppress T cell proliferation through modulation of arginine and cysteine availability and production of nitric oxide and reactive oxygen species [22, 23]. In addition to their immunosuppressive functions, MDSCs can also promote angiogenesis, and have even been shown to become incorporated into the endothelium [24]. Furthermore, MDSCs within the bone microenvironment can differentiate into functional osteoclasts capable of degrading bone [25, 26]. They have also been implicated in the formation of a pre-metastatic niche, creating a microenvironment in distant organs that is favorable for tumor growth [27, 28]. Taken together, this suggests that MDSCs have important roles in promoting metastatic outgrowth in the bone microenvironment.

A proteomic study recently demonstrated that cathepsin B expression in MDSCs isolated from mice bearing 4T1 tumors was three-fold increased compared to cells isolated from mice bearing 67NR mammary tumors [29]. Cathepsin activity was also shown to be abundant in tumor cells and MDSCs isolated from 4T1 mammary tumors [30]. These data led to the hypothesis that cathepsins derived from MDSCs, in addition to tumor cells, play pro-metastatic roles. We have utilized quenched activity-based probes in combination with cathepsin-specific inhibitors to carefully assess cathepsin levels and function within the tumor microenvironment. We show that MDSC-derived cathepsin activity decreases during osteoclastogenesis and that cathepsin inhibitors promote osteoclast differentiation. Further, we show that inhibition of legumain, a protease known to promote cathepsin activity, has a similar effect. Together, this suggests that cysteine cathepsin proteases have a suppressive role in osteoclasteogenesis, a very important process in bone metastasis.

## RESULTS

### Cysteine cathepsin levels are increased in mice bearing metastases

**Figure 1:**
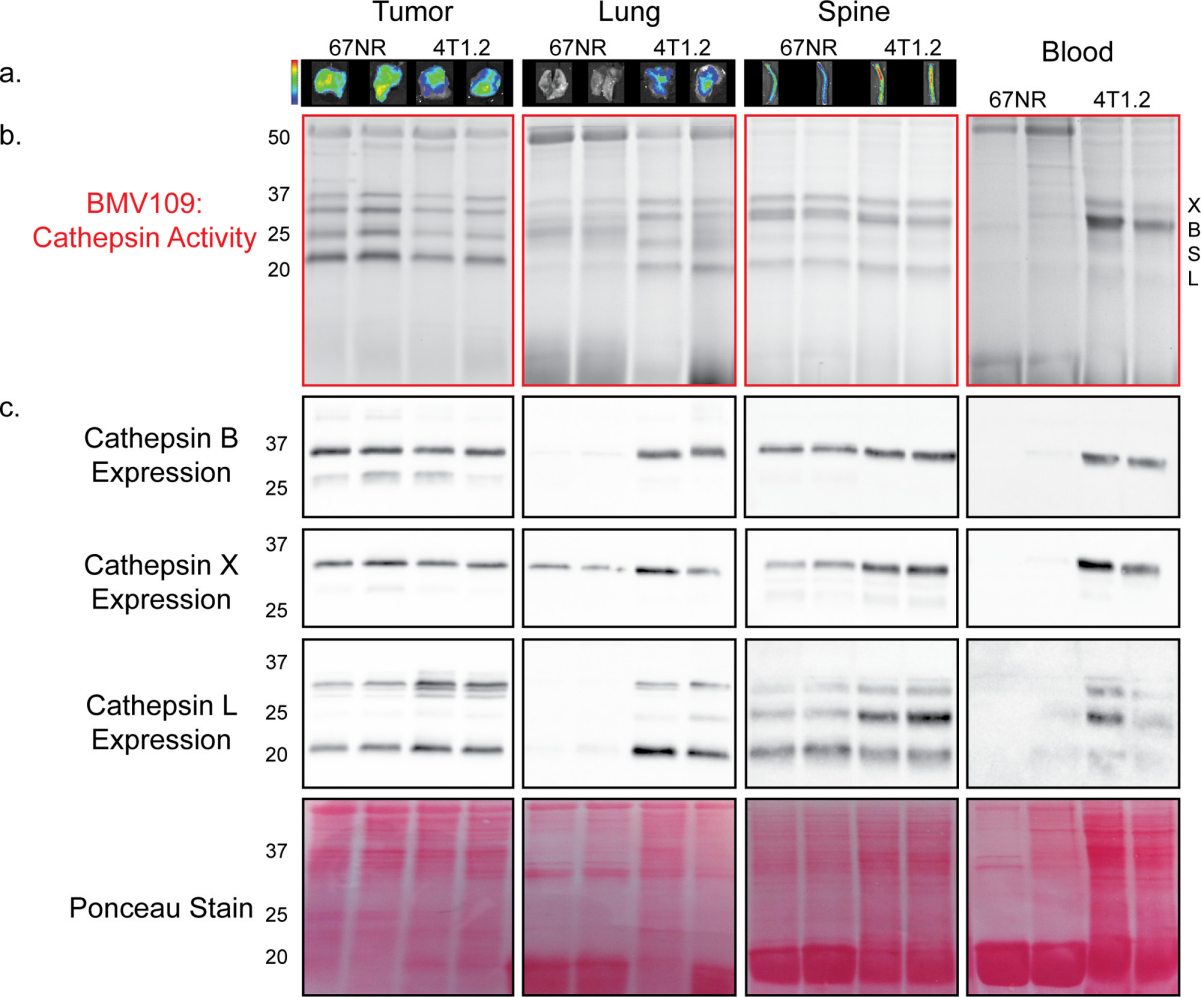
*In vivo* characterization of cysteine cathepsin levels in tissues from tumor-bearing mice **a.** Mice bearing 67NR or 4T1.2 primary tumors were injected with BMV109, and primary tumors, lungs and spines were imaged *ex vivo* for fluorescence due to cathepsin activity. Minimum and maximum values were set for each tissue type as follows [(p/sec/cm^2^/sr)/(μW/cm^2^)]: Tumor 4e8–1.4e9; Lung 3e8–9e8; Spine 2.7e8–9.2e8. **b–c.** Tissues in (a) as well as peripheral blood mononuclear cells were lysed and analyzed by SDS-PAGE. (b) BMV109 labeling indicates cathepsin activity (top panel) while (c) western blots with cathepsin-specific antibodies indicate expression (bottom panels). Darker bands indicate higher activity/expression. Two representative samples are shown for each tissue. Ponceau staining was used to ensure that equal protein was loaded.

To examine metastasis-dependent changes in cysteine cathepsin activity, we utilized the quenched activity-based probe, BMV109 [19]. Quenched activity based-probes are small molecules that are intrinsically dark and emit fluorescence only when cleaved by proteases, thereby reporting levels of protease activity [31]. BMV109 is a pan-cysteine cathepsin probe that allows for simultaneous monitoring of cathepsins X, B, S, and L [19]. Breast cancer cells, either 67NR (non-metastatic) or 4T1.2 (metastatic), were injected in the fourth mammary gland of mice. When signs of lung and bone metastasis were evident in the 4T1.2 mice (labored breathing and/or paralysis; approximately 25 days), we injected BMV109 intravenously. Six hours later, tissues were harvested and imaged *ex vivo* for cathepsin-dependent fluorescence (Figure 1a). We observed similar levels of cathepsin activity in 67NR and 4T1.2 primary tumors (Figure 1a). Tissues bearing 4T1.2 metastases (lung and spine), however, exhibited increased activity (Figure 1a).

To determine exactly which cysteine cathepsins were contributing to the *ex vivo* fluorescence, the tissues were lysed and analyzed by fluorescent SDS-PAGE. We observed several bands corresponding to active cathepsin X, B, S, and L (Figure 1b). The identity of these bands was confirmed by immunoprecipitation with cathepsin-specific antibodies (Supplementary Figure S1a). We also performed western blots on these tissue lysates to survey total cathepsin expression. Cathepsin X, B, S, and L were expressed to similar extents in 67NR and 4T1.2 primary tumors (Figure 1c). In contrast, lungs with 4T1.2 metastases exhibited a strong increase in cathepsin expression/activity compared to lungs from mice bearing non-metastatic 67NR tumors (Figure 1a–1c). This was also observed in the spine, but to a lesser extent, which is in line with a lower metastatic burden in bone. Surprisingly, we also observed a substantial increase in the activity and expression of cathepsin X, B, and L in mononuclear cells isolated from the peripheral blood of mice with metastases (Figure 1b–1c). This indicates that cathepsin activity is systemically upregulated during metastasis.

### Cysteine cathepsins are active in myeloid-derived suppressor cells

**Figure 2:**
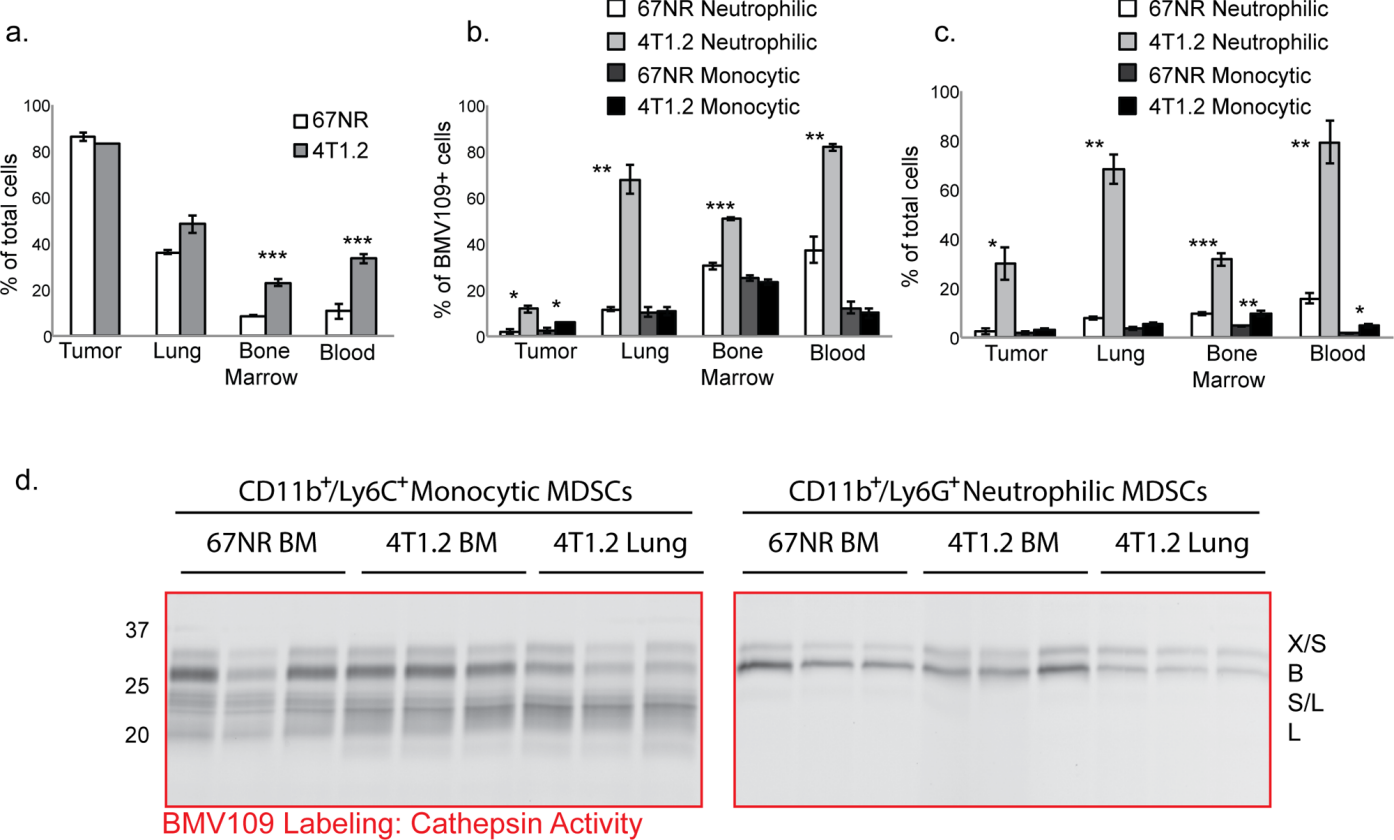
MDSCs produce active cysteine cathepsins BMV109-labeled tissues were dissociated into single cell suspensions and analyzed by flow cytometry. **a.** Comparisons of the percentages of BMV109^+^ cells in tissues from 67NR and 4T1.2 tumor-bearing mice. Error bars represent SEM. **b.** The percentage of the BMV109^+^ cells in (a) that were positive for CD11b and Ly6G (neutrophilic MDSC markers) or CD11b and Ly6C (monocytic MDSC markers). **c.** The percentage of all cells that are neutrophilic or monocytic MDSCs. For (a-c) asterisks indicate statistical significance between the percentage of 67NR and 4T1.2 cells within each subset. *p < 0.05, **p < 0.01, ***p < 0.001. **d.** Cathepsin activity in sorted neutrophilic and monocytic MDSCs from bone marrow and lungs of 67NR or 4T1.2 tumor-bearing mice. Darker bands indicate higher activity.

We next used flow cytometry to assess levels of cathepsin activity in tissues obtained from metastatic and non-metastatic mice injected with BMV109. The proportion of BMV109^+^ cells was similar in 67NR and 4T1.2 primary breast tumors; however, in lung, bone marrow, and blood of mice bearing metastases, this proportion was increased (Figure 2a). A large percentage of the cells producing active cathepsins were myeloid-derived suppressor cells of both neutrophilic (CD11b^+^/Ly6G^+^) and monocytic (CD11b^+^/Ly6C^+^/Ly6G^−^) subsets (Figure 2b). Both of these populations were dramatically expanded in tissues from mice with metastasis; however, the neutrophilic subsets were considerably more abundant (Figure 2c & Supplementary Figure S2).

To identify precisely which cysteine cathepsins are active in MDSCs, we also sorted cells from tissues by flow cytometry and labeled them with BMV109 *ex vivo*. CD11b^+^/Ly6G^+^ neutrophilic MDSCs produced active cathepsin X and B, whereas CD11b^+^/Ly6C^+^/Ly6G^−^monocytic MDSCs contained active S and L in addition to X and B (Figure 2d & Supplementary Figure S1b). Cathepsin activity in MDSCs derived from bone marrow of 67NR and 4T1.2 mice were comparable (Figure 2d). Interestingly, however, cathepsin B levels were substantially higher in MDSCs isolated from the bone marrow of 4T1.2 mice compared to those taken from lungs (Figure 2d). This was observed in both MDSC subsets. These results suggest differential regulation of MDSC-derived cathepsin B within the bone microenvironment. While cathepsin levels in bone marrow MDSCs from metastatic and non-metastatic mice are not different on a per cell basis, the number of MDSCs in the 4T1.2 mice is vastly higher (Figure 2c). Hence, given the abundance of MDSCs in 4T1.2 mice, there is substantially higher cathepsin activity in the bone microenvironment.

### Cysteine cathepsins do not contribute to immune suppression by MDSCs

Considering it was clear that cathepsins were highly expressed in MDSCs, we next examined whether cathepsin activity could contribute to the canonical immunosuppressive functions of MDSCs. Splenocytes from naïve mice were labeled with CFSE and cultured in the presence and absence of Gr-1^+^ (Ly6C^+^/Ly6G^+^) cells that were isolated from blood or bone marrow of mice bearing 4T1.2 experimental bone metastases. When stimulated with CD3 and CD28, CD4^+^ and CD8^+^ T cells exhibited substantial proliferation, as evidenced by dilution of their CSFE label (Supplementary Figure S3). Proliferation was reduced in the presence of blood MDSCs, and this was even more pronounced with bone marrow MDSCs. Neither T cell proliferation nor MDSC suppression was altered by the addition of cathepsin inhibitors (CA074: cathepsin B-selective inhibitor [32] or JPM-OEt: pan cathepsin inhibitor [33]). These data suggest that cathepsins do not contribute to T-cell specific immunosuppressive functions of MDSCs.

### Inhibitory role for cysteine cathepsins during osteoclast differentiation

**Figure 3:**
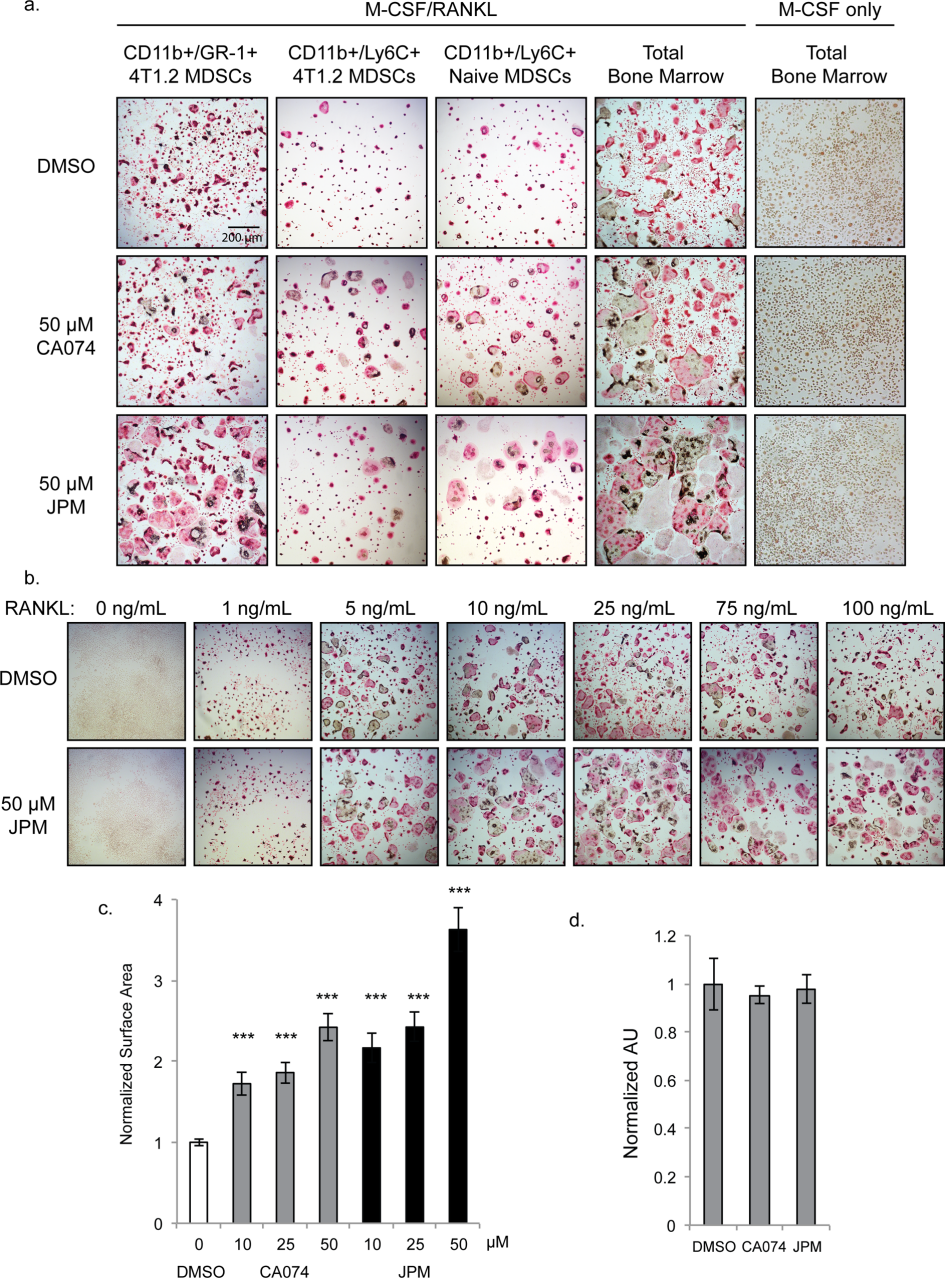
Cysteine cathepsin inhibition promotes osteoclast fusion **a.** TRAP staining of osteoclasts differentiated with M-CSF/RANKL in the presence of 50 μM CA074, JPM-OEt or DMSO vehicle control. CD11b^+^/Gr-1^+^ 4T1.2 MDSCs and CD11b^+^/Ly6C^+^ 4T1.2 MDSCs were isolated from mice with experimental 4T1.2 metastasis. CD11b^+^/Ly6C^+^ Naïve MDSCs and total bone marrow were harvested from naïve, non-tumor-bearing mice. Bone marrow cells cultured without RANKL (rightmost column) did not form osteoclasts. **b.** Dose response of RANKL treatment of naïve total bone marrow cells stimulated with M-CSF and either DMSO or 50 μM JPM-OEt. **c.** Quantification of surface area of osteoclasts differentiated with increasing doses of CA074 or JPM-OEt. > 250 osteoclasts were measured for each condition. ***p < 0.001 **d.** SRB proliferation assay of naïve bone marrow cells cultured with M-CSF for 4 days. Error bars represent SEM.

Given the increased cathepsin B activity in MDSCs derived from bone marrow compared to lung, we next examined whether cathepsin activity was required for the differentiation of MDSCs into multinucleated osteoclasts. CD11b^+^/Gr-1^+^ MDSCs isolated from the bone marrow of mice bearing 4T1.2 bone metastases were stimulated with RANK ligand (RANKL) and M-CSF to undergo osteoclastogenesis. Unexpectedly, osteoclast formation was significantly enhanced when cells were differentiated in the presence of the cathepsin-B specific inhibitor, CA074, and even more strikingly with JPM-OEt, the pan-cathepsin inhibitor (Figure 3a). CD11b^+^/Ly6C^+^/Ly6G^−^ monocytic MDSCs exhibited a similar response to cathepsin inhibition, whether isolated from tumor-bearing or naïve bone marrow (Figure 3a). CD11b^+^/Ly6G^+^ neutrophilic MDSCs, on the other hand, did not form osteoclasts (not shown). Differentiation of total bone marrow from naïve mice was also enhanced by cathepsin inhibition (Figure 3a), and this was observed at both high and low concentrations of RANKL (Figure 3b). To quantify this enhancement, we measured osteoclast surface area and found that the average cell size was significantly increased by cathepsin inhibitors in a dose-dependent manner (Figure 3c).

Cathepsin inhibition could influence osteoclast size in one of two ways: 1) increasing proliferation rates of precursor cells allowing more fusion events to occur or 2) enhancing the rate of fusion itself. To investigate the former possibility, we examined proliferation rates of cells cultured in the presence of M-CSF alone. Cathepsin inhibition did not significantly impact the density of precursor cells differentiated with M-CSF only as shown by TRAP staining (Figure 3a) and SRB proliferation assay (Figure 3d). Differences between control and cathepsin-inhibited cells were not evident until day 4 of differentiation, which further indicates that this effect was not due to enhanced proliferation, but rather, enhanced fusion of precursor cells.

**Figure 4:**
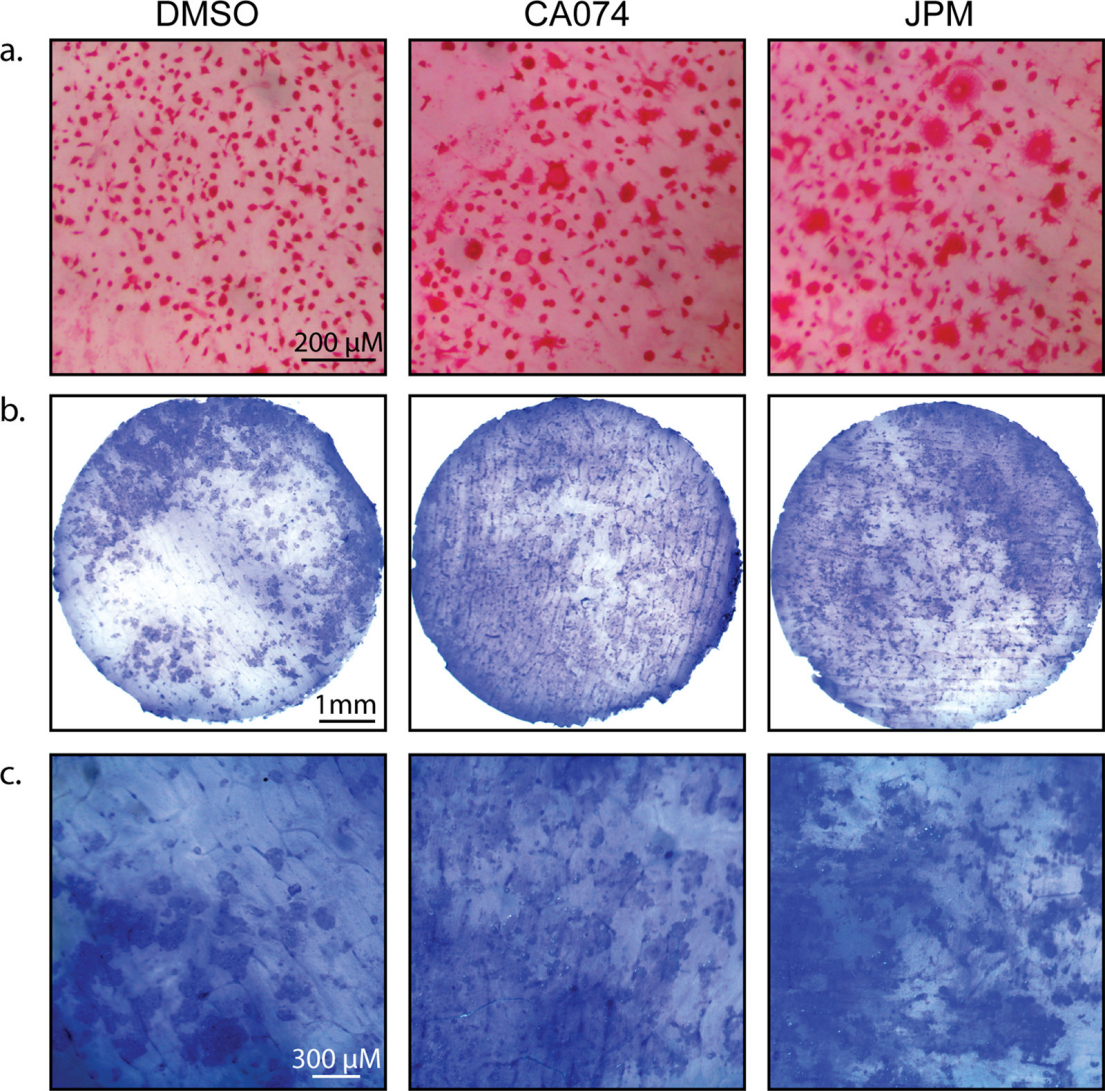
Cysteine cathepsin inhibition enhances osteoclast activity on bone slices **a.** CD11b^+^/Gr-1^+^ MDSCs were seeded on bovine cortical bone slices and stimulated with M-CSF and RANKL in the absence and presence of CA074 or JPM-OEt. After 7 days, cells were stained for TRAP activity. **b.** Total bone marrow cells were seeded on bovine cortical bone slices and stimulated with M-CSF and RANKL in the absence and presence of CA074 or JPM-OEt. On day 13, osteoclasts were removed and bone slices were stained with toluidine blue to visualize pit formation/bone degradation by osteoclasts. **c.** A higher magnification of bone slices depicted in (b).

Increased differentiation/fusion by cathepsin inhibition was also observed when osteoclasts were differentiated on bovine cortical bone slices (Figure 4a), and importantly, these cells were functional in bone degradation assays (Figure 4b–4c).

### Cysteine cathepsin activity is naturally downregulated during osteoclastogenesis

**Figure 5:**
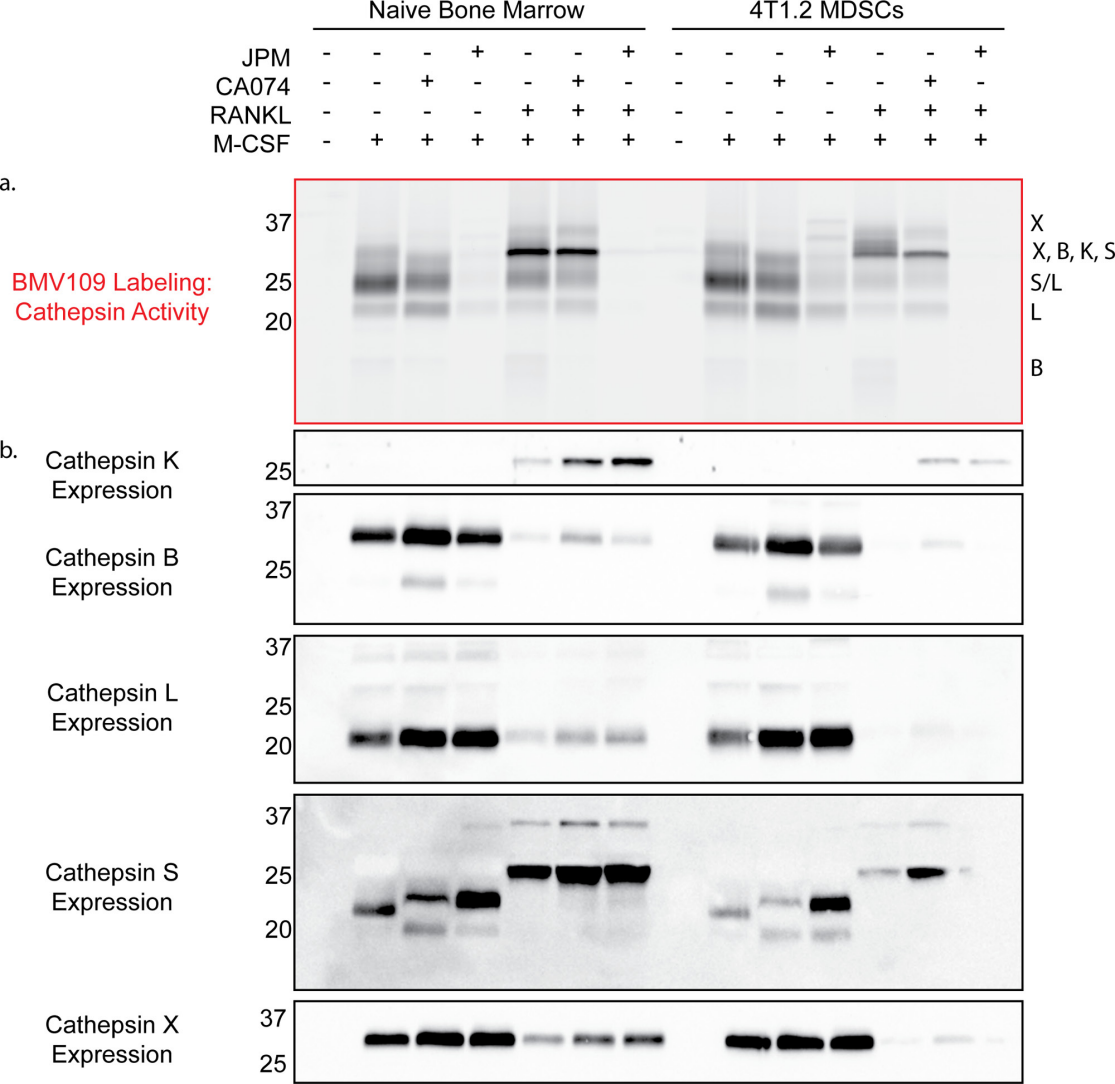
Biochemical analysis of cysteine cathepsin activity and expression in precursors and mature osteoclasts **a.** Cathepsin activity and **b.** expression in unstimulated cells, macrophage precursors stimulated with M-CSF only, and mature osteoclasts stimulated with M-CSF and RANKL. The left side depicts cells differentiated from total bone marrow of naïve mice, while the right side is CD11b^+^/Gr-1^+^ MDSCs from mice with 4T1.2 experimental metastasis. Darker bands indicate higher activity/expression.

**Figure 6:**
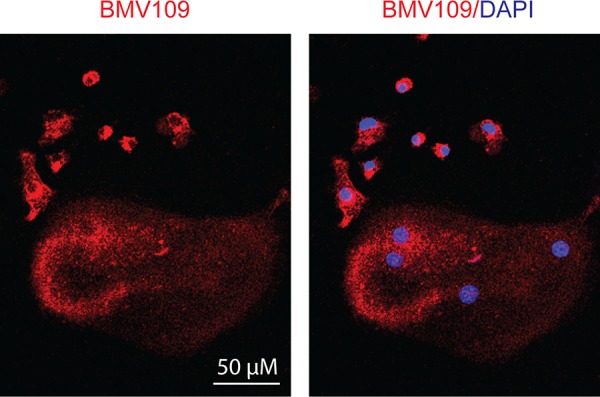
Cysteine cathepsin activity is downregulated in mature osteoclasts compared to macrophage precursors Osteoclasts were differentiated in the presence of M-CSF and RANKL, labeled with BMV109, and imaged by confocal fluorescent microscopy. Red indicates cathepsin activity and blue depicts DAPI staining of nuclei.

Next we performed a biochemical analysis of cathepsin activity and expression during osteoclastogenesis in both MDSC and total bone marrow cultures. Cells were analyzed immediately after harvesting or seven days after stimulation with either M-CSF alone (macrophage precursors) or M-CSF/RANKL (mature osteoclasts). During the transition from naïve cells to macrophages, activity of cathepsin X, B, S, and L was strongly upregulated (Figure 5a & Supplementary Figure S1c). As expected, differentiation to osteoclasts was marked by a sharp increase in cathepsin K activity/expression (Figure 5a–5b). Expression of cathepsin B, L, and X was concomitantly decreased (Figure 5b). This suggests that these cathepsins are naturally downregulated during differentiation from macrophages to osteoclasts, supporting an inhibitory function during osteoclastogenesis. This was also evident in fluorescent images of cells labeled with BMV109. Cathepsin signal was lower and more diffuse in osteoclasts than in mononuclear precursor cells in the same culture (Figure 6). Cathepsin S, on the other hand, was not downregulated, but present as two higher molecular weight species, indicating that this protease is differentially processed in osteoclasts (Figure 5b).

Labeling of cathepsin B by BMV109 was uniquely blocked by treatment with the cathepsin B-specific inhibitor CA074, while nearly all cathepsin activity was abrogated when cells were treated with the pan-cathepsin inhibitor, JPM-OEt (Figure 5a). Cathepsin K activity was inhibited by JPM-OEt but not CA074, yet its total expression was increased by both inhibitors (Figure 5a–5b), corresponding to the enhanced osteoclastogenesis we observed by TRAP staining (Figure 3a). Cathepsin X, B, S, and L were also slightly upregulated in the presence of the inhibitors, reflecting the compensatory nature of the regulation of these proteases (Figure 5b). We also treated cells with CA074-methyl ester (CA074me), which is known to have increased cell permeability over CA074. While CA074me increased osteoclast size in a dose-dependent manner, it was by no means cathepsin B-specific (Supplementary Figure S4a–S4c), as observed by competition of labeling of cathepsin X, S, L, and K.

### Legumain inhibitors alter cathepsin expression in osteoclasts

**Figure 7:**
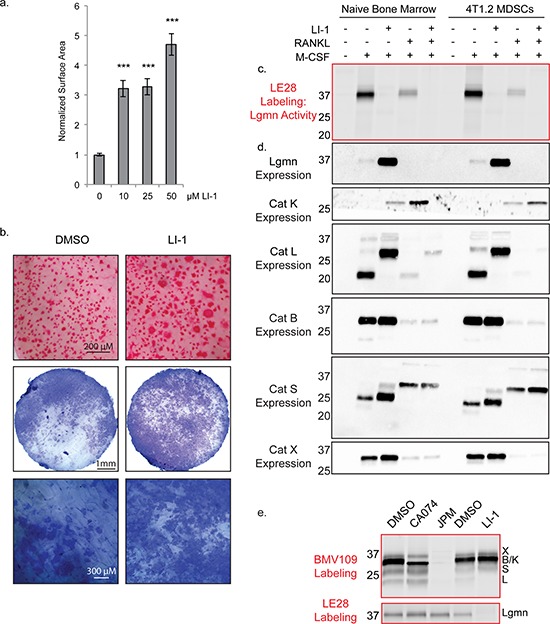
Legumain inhibitors promote osteoclastogenesis through modulation of cathepsin L activation **a.** Average surface area of osteoclasts differentiated with increasing concentrations of the legumain inhibitor LI-1. > 250 cells were measured for each condition. Error bars represent SEM. ***p < 0.001 **b.** TRAP staining on bone and pit assay of osteoclasts differentiated with LI-1 or DMSO control. **c.** Biochemical assessment of legumain activity by LE28 labeling as well as **d.** western blots depicting expression of legumain and cathepsins. **e.** BMV109 labeling (cathepsin activity) and LE28 labeling (legumain activity) in osteoclasts treated with cathepsin and legumain inhibitors. Darker bands indicate higher activity/expression.

Legumain (asparaginyl endopeptidase) is a related cysteine protease that has been previously shown to inhibit osteoclastogenesis through autocleavage of its C-terminal domain [34, 35]. As we have demonstrated for cathepsin protein, legumain mRNA levels decrease as osteoclastogenesis progresses [35]. Since legumain can regulate cathepsin activity [36], we wondered whether the legumain-specific inhibitor LI-1 [37] would affect cathepsin activation during osteoclastogenesis. LI-1 dramatically increased the surface area of osteoclasts, to an even greater extent than any of the cathepsin inhibitors (Figure 7a). Osteoclasts were also larger in the presence of LI-1 when cultured on bone slices, and these cells were functional in pit assays (Figure 7b).

We also utilized the quenched activity-based probe LE28 [38] to biochemically monitor legumain activity in macrophage precursors and in mature osteoclasts. Legumain activity was decreased in mature osteoclasts compared to macrophages, and was completely blocked upon addition of LI-1 (Figure 7c & Supplementary Figure S1d). Reducing legumain activity resulted in a dramatic compensation of expression in macrophages; however, in osteoclasts, expression was below the threshold of detection for the antibody (Figure 7d). Cathepsin K expression was increased upon legumain inhibition; however, cathepsin X, B, and S were only mildly affected (Figure 7d). Cathepsin L, on the other hand, was exclusively expressed as a high molecular weight species when LI-1 was added (Figure 7d). When cells were labeled with the BMV109 cathepsin probe after LI-1 treatment, cathepsin L activity was not detected (Figure 7e). Conversely, cathepsin inhibition had no impact on legumain activity (Figure 7e). This suggests that legumain is responsible for cleavage and activation of cathepsin L in osteoclasts. Legumain inhibitors may therefore regulate osteoclastogenesis by blocking legumain activity directly as well as through modulation of cathepsin activity.

## DISCUSSION

Cysteine cathepsins have been widely studied in the context of cancer; however, their site-specific functions are only just beginning to be understood [39]. Joyce and colleagues have elegantly demonstrated that the cellular source of proteases can impact its function. Cathepsins supplied by primary tumor cells and tumor-associated macrophages function in unique ways [8, 9]. To further complicate the picture, some cathepsins have distinct substrates at sites of secondary tumor growth, giving rise to additional, organ-specific functions [4]. As such, a systemic assessment of the tumor and stromal function of cathepsins is required for developing therapies based on cathepsin inhibition.

We used a biochemical approach to simultaneously monitor the activity and expression of cathepsins X, B, S, and L in a mouse model of breast cancer metastasis. Western blotting allowed us to examine the regulation of cathepsins at the expression level, and the use of the BMV109 activity-based probe revealed a deeper understanding of the post-translational regulation of the active pools of enzymes. Cysteine cathepsin levels were systemically increased in 4T1.2-bearing mice compared to those with 67NR tumors, both at distant sites of metastasis and in peripheral blood.

Myeloid-derived suppressor cells have been identified as important promoters of metastasis in mouse models of breast cancer and in human patients [16]. MDSCs have been previously identified as cathepsin-producing cells [10, 29, 30]. In a proteomic study, cathepsin B expression was reported to be increased in CD11b^+^/Gr-1^+^ MDSCs from spleens of mice bearing 4T1 compared to 67NR tumors [29]; however, no indication of the functional, active, pool of cathepsin B was previously reported. We used BMV109 to examine the activity of cathepsins within neutrophilic and monocytic MDSC populations isolated from lung and bone marrow, the most common sites of metastasis. While the cathepsin profile differed between the two subsets, we did not observe significant changes in activity between cells isolated from the bone marrow of metastatic and non-metastatic mice. The amount of protein that we were able to obtain from the sorted MDSCs was below the threshold of detection for the antibody; however, if total expression was indeed increased as the proteomics experiment suggests, the impact on activity may be negated by increased levels of endogenous cathepsin inhibitors in metastatic MDSCs identified in the same study [29]. Further investigation of the relationship between cathepsin and cystatin regulation within MDSCs is of ongoing interest. While levels of active cathepsins in the bone marrow were not affected by metastasis on a per cell basis, the number of cathepsin-expressing cells was greatly increased by the presence of 4T1.2 outgrowths. There is no question that the amount of MDSC-derived cathepsin activity within the bone tumor microenvironment was increased.

Since cathepsins can be secreted into the extracellular space, MDSC-derived cathepsins may impact tumor cell growth and invasion or have direct effects on MDSC function. We saw no contribution of cathepsin activity to immunosuppression of T cell activation by MDSCs; therefore, we focused on the bone-specific functions of MDSCs. Previous studies describing the osteoclastogenic potential of MDSCs [25, 26] have used CD11b^+^/Gr-1^+^ populations, which encompasses both the Ly6G^+^ (neutrophilic) and Ly6C^+^ (monocytic) MDSCs. As we have observed in this study and others, these subsets are quite heterogeneous in their function. The neutrophilic MDSCs simply did not form osteoclasts, while the monocytic ones did. While the monocytic cells may be a small proportion of the total “MDSC” pool, they have unique functions and are likely playing important roles in the bone tumor microenvironment. It is quite likely that cathepsins are important for the function of neutrophilic MDSCS as well; however, their roles in this context are still poorly understood. Other MDSC-derived proteases such as matrix metalloproteases have been shown to be important for promoting angiogenesis within the tumor microenvironment [24]. The serine protease uPA produced by tumor cells contributes to MDSC recruitment into the tumor microenvironment [40]. It is possible that cysteine cathepsins, and likely other families of proteases, may be important for these functions as well.

Based on existing literature investigating the contribution of cathepsins during macrophage-to-osteoclast differentiation, we initially hypothesized that cathepsin inhibition would prevent MDSC precursors from becoming osteoclasts. The endogenous cathepsin inhibitor Cystatin C, as well as non-selective chemical inhibitors CA-074me and E64, have been shown to inhibit osteoclast formation and bone resorption [41–44]. In our hands, we were quite surprised to see that, not only did osteoclasts form in the presence of cathepsin inhibitors, they were larger and more functional than control-treated MDSCs. These larger osteoclasts also expressed higher levels of the osteoclast-specific protease, cathepsin-K. The increased osteoclastogenesis observed after cathepsin inhibition was not MDSC- or cancer-dependent, as cathepsin inhibition had the same effect on osteoclastogenesis of total bone marrow from naïve Balb/c mice. This observation was reproduced in multiple experiments using multiple inhibitors at a range of RANKL concentrations. We cannot explain the inconsistency of our results with the published studies, but it may be down to differences in mouse strains, sources of cytokines, or isolation/differentiation protocols. In any case, we have shown that cells can indeed differentiate into osteoclasts without the activity of the major cathepsin players. In support of our results, bone marrow macrophages from cathepsin B null mice do not have reduced osteoclastogenic potential [43]. Previous inhibitor studies have lacked follow-up biochemistry with activity-based probes, whereas we have confirmed inhibition of target proteases by multiple inhibitors. The fact that legumain also inhibits osteoclast differentiation and is known to increase cathepsin activity supports our findings that cathepsins inhibit, rather than accelerate, osteoclastogenesis. While our evidence conflicts with the above-mentioned reports, we feel it is important to highlight given the impact of osteoclastogenesis on bone metastasis and the fact that targeting cathepsin B or other cathepsin proteases could actually have detrimental effects by promoting the “vicious cycle” of bone degradation and tumor growth.

Our analyses have also revealed complex regulation of cathepsin expression and activity during the transition from MDSC/macrophage precursors to osteoclasts. Previous studies have suggested that cathepsin K is the dominant cathepsin in osteoclasts [45] and that B, L, and S mRNA levels are 20-, 130- and 400-fold lower, respectively [46]; however, we have shown that they are present and proteolytically active in detectable quantity. While cathepsin K is strongly upregulated during the transition from MDSCs/macrophages to osteoclasts, cathepsin X, L, and B expression and activity are downregulated. This observation agrees with previous data demonstrating that cathepsin B mRNA levels are decreased in mature osteoclasts [43]. By contrast, cathepsin S expression is not downregulated but it is present in osteoclasts as a less mature species. These data indicate that cathepsin activity is naturally downregulated during osteoclastogenesis, and that cathepsin inhibitors or, indirectly, legumain inhibitors further augment this effect. We hypothesize that cathepsin activity may normally inhibit fusion of osteoclast precursors through cleavage of proteins that are required for membrane fusion. One potential target is E-cadherin, a well-known cathepsin substrate [9]. Antibodies blocking E-cadherin negatively impact osteoclast fusion [47] as does a dominant negative form of N-cadherin [48]. Reduced cathepsin activity may consequently increase cadherin levels, resulting in enhanced osteoclast fusion. The delayed onset of the effect of cathepsin inhibitors (after day four) and their lack of impact on precursor proliferation provide further support that this is a fusion-mediated effect.

Increased osteoclast activity in the bone tumor microenvironment has been associated with enhanced metastasis and bone degradation, and osteoclasts may have roles in promoting outgrowth from tumor cell dormancy [49]. Many groups, including ours, have suggested that targeting specific cathepsins is a valid therapeutic approach in breast cancer [6, 7, 50]. Our current study supports a possible bone metastasis suppressive function of MDSC-derived cathepsins and legumain, and this should be further investigated before considering such therapies as anti-metastatic approaches.

## MATERIALS AND METHODS

### Cell lines

4T1.2-luc2-mCherry and 67NR-mCherry cells were developed by Robin Anderson's laboratory at the Peter MacCallum Cancer Centre, originally from the Miller series of cell lines [13–15]. Both lines were maintained in Minimal Essential Media (alpha modification) + L-glutamine (MEM alpha; Gibco) supplemented with 5% fetal bovine serum and 1% penicillin/streptomycin at 37°C with 5% CO_2_. For passaging, cells were washed twice with PBS and lifted with 0.01% EDTA at 37°C.

### Mouse models

All animal experiments were approved by the La Trobe University Animal Ethics Committee.

### Spontaneous metastasis model

All experiments were performed on 7-week old female Balb/c mice purchased from the Walter & Eliza Hall Institute. Tumor cells (100,000 in 20 μl PBS) were injected into the fourth mammary fat pad of mice anesthetized with isoflurane. Upon signs of metastasis (day 21–27), tissues were harvested for MDSC analysis. Alternatively, mice were intravenously injected with the activity-based probe, BMV109 (5 nmol in 100 μl of 20% DMSO/PBS). After six hours, tissues were harvested and imaged for Cy5 fluorescence on an IVIS Lumina-XR III *in vivo* imaging system (Perkin Elmer). Tissues were then divided for further analysis.

### Experimental metastasis model

Mice were anesthetized using isoflurane and tumor cells (30,000 in 100 μl) were injected into the left cardiac ventricle using a 26-gauge needle. Signs of bone metastasis became evident after 10–13 days, at which point, bones were harvested to obtain MDSCs.

### Flow cytometry and MDSC isolation

Tumors and lungs were minced with a razor blade followed by digestion with collagenase (1 mg/mL) and DNaseI (30 μg/mL) in RPMI with 5% FBS for 1.5 hours at 37°C. Bone marrow was obtained by flushing bones with FACS buffer (2% FBS, 1% pen/strep in PBS). Single cell suspensions were subject to erythrocyte depletion with red blood cell lysis buffer (150 mM NH_4_Cl, 1 mM KHCO_3_, 0.1 mM EDTA) followed by two washes with FACS buffer. Cells were then stained with the indicated antibodies for 10 minutes at room temperature before flow cytometry analysis. [rat anti-mouse Gr-1-PerCP (Biolegend, #108426, clone RB6-8C5); rat anti-mouse CD11b-FITC (BD Biosciences, #553310, clone M1/70); rat anti-mouse Ly6G-PE (BD Biosciences, #551461, clone 18A); rat anti-mouse Ly6C-APC (eBioscience, # 17-5932-82, clone HK1.4)] Sorting experiments were performed on a BD FACSAriaIII cell sorter and all others were analyzed on a BD FACSCantoII. Analysis was performed using FlowJo software. Average percentages were reported with error bars representing standard error of the mean.

### T cell suppression assay

Blood and bone marrow were harvested from mice 10 days after intracardiac injection with 4T1.2 tumor cells. After erythrocyte depletion, cells were washed with FACS buffer and rat anti-mouse Gr-1 antibody (eBioscience, # 14-5931-82, clone RB6-8C5) was added at 5 μg/mL for 30 minutes on ice. Unbound antibody was removed by washing twice with FACS buffer, and BioMag goat anti-rat IgG magnetic beads (Qiagen) were added at 10:1 bead-to-cell ratio. Tubes were nutated for 30 min at 4°C followed by 3 washes with FACS buffer with the assistance of a BioMag Magnet. Bead-bound cells were then analyzed for purity by flow cytometry and were shown to be >95% CD11b^+^. Total splenocytes were obtained from a naïve mouse by mechanical dissociation with a 100-μm cell strainer. Cells were labeled with carboxyfluorescein succinimide ester CFSE (1 μM) for eight minutes at room temperature followed by two washes with FBS-containing media. Labeled splenocytes (100,000) were then stimulated to proliferate with MEM alpha medium supplemented with 5% FBS, CD3 (200 ng/mL) and CD28 (1 μg/mL). Gr-1^+^ MDSCs (100,000) were added where indicated in the presence of cathepsin inhibitors (50 μM) or DMSO vehicle. After a 72-hour incubation at 37°C, cells were labeled with antibodies for CD4 and CD8: rat anti-mouse CD4-APC (eBioscience, # 17-0042-82, clone RM4-5); rat anti-mouse CD8-APC-eFluor780 (eBioscience, #47-0081-82, clone 53.6-7). T cells were then analyzed for loss of CFSE by flow cytometry.

### Protease labeling and western blotting

Cells were washed with PBS and lysed by freezing in citrate buffer (50 mM citrate, pH 5.5, 0.5% CHAPS, 0.1% Triton X-100, 4 mM DTT). Supernatants were cleared by centrifugation. Total protein concentration was determined by BCA assay (Pierce). Activity-based probes [BMV109 (0.1 μM) or LE28 (1 μM)] were added to lysates from a 100x stock, and proteins were incubated at 37°C for 45 minutes [51]. Reaction was stopped by addition of 4x sample buffer (40% glycerol, 200 mM Tris-Cl, pH 6.8, 0.04% bromophenol blue, 5% beta-mercaptoethanol) followed by boiling for five minutes. Total protein (30–50 μg) was resolved by SDS-PAGE (15%). Gels were then scanned for Cy5 fluorescence using a Typhoon flatbed laser scanner (GE Healthcare). Where required, gels were transferred to nitrocellulose membranes using a Trans-Blot Turbo Transfer System (BioRad) and subject to standard western blotting protocols. Antibodies: goat anti-cathepsin X (1:1000; R&D AF1033), goat anti-cathepsin B (1:1000; R&D AF965), goat anti-cathepsin L (1:1000; R&D AF1515), sheep anti-legumain (1:1000; R&D AF2058), goat anti-cathepsin S (1:500; Abcam 18822), goat anti-cathepsin K (1:500; Abcam 19027). Tissues labeled with BMV109 *in vivo* were lysed by sonication in citrate buffer, and then equal protein was analyzed by fluorescent SDS-PAGE as above.

### Immunoprecipitations

Probe-labeled lysate was divided into input and IP samples (~50 μg each) [51]. IP samples were diluted in 500 μl IP buffer (PBS, pH 7.4, 0.5% NP-40, 1 mM EDTA), and 5 μl of the appropriate antibody was added followed by a 10-minute incubation on ice. Protein A/G beads (40 μl slurry; Santa Cruz Biotechnology) were then washed with IP buffer and added to the samples. After rocking overnight at 4°C, beads were washed four times with IP buffer and once with 0.9% sodium chloride. 2x sample buffer (20 μl) was added, and beads were boiled. Supernatants were resolved by SDS-PAGE alongside input sample, and the gel was scanned for Cy5 fluorescence using a Typhoon scanner.

### Osteoclast assays

MDSCs were isolated as described above. For experiments using total bone marrow, femurs were flushed with complete MEM alpha medium. Cells were resuspended in red blood cell lysis buffer (150 mM NH_4_Cl, 1 mM KHCO_3_, 0.1 mM EDTA) to deplete erythrocytes and then washed with media. Cells were then cultured in media in a T75 flask at 37°C. The next day, non-adherent cells were counted and used without further purification. To stimulate osteoclastogenesis, MDSCs or total bone marrow were seeded in 96-well plates at 50,000 cells per well in 100 μl complete MEM alpha medium containing RANKL (1–100 ng/mL) and/or M-CSF (50 ng/mL) [26]. Inhibitors or DMSO vehicle were added at the indicated concentration at the time of stimulation with final DMSO concentrations not exceeding 0.04%. Fresh medium containing RANKL, M-CSF and inhibitors (30 μl) was added every two days until mature osteoclasts formed (five to eight days). TRAP staining was then performed as described below.

### TRAP staining

Cells were washed three times with PBS and then fixed for 20 minutes at room temperature with 100 μl acetate buffer (110 mM sodium acetate, 50 mM sodium tartrate dibasic dehydrate, 0.28% glacial acetic acid, pH 4.8). Napthol AS-BI Phosphate Substrate (44 mM in 2-ethoxyethanol) was diluted in acetate buffer at a ratio of 1:100, and 100 μl of this solution was added to each well for 1 hour at 37°C. Sodium nitrite (0.58 M) and pararosaniline dye (154 mM in 2M HCl) were combined 1:1 and then diluted in acetate buffer at a ratio of 1:25. The napthol solution was removed from the cells, and 100 μl of the pararosaniline solution was added directly without any washing step. Cells were incubated at 37°C for 5–10 minutes until a red stain developed. The reaction was stopped with three washes in distilled water and then stored in water at 4°C. Bright-field images were obtained using a 4x objective on a Nikon Ti Eclipse epi-fluorescence microscope.

### Pit assay

Osteoclasts were cultured as above except on bovine cortical bone slices (Immunodiagnostic Systems) instead of plastic. On day 7, bone slices were TRAP stained as above. For functional analysis, osteoclasts were removed by mechanical dislodgement with a cotton bud on day 13. Bones were then stained for 10 seconds in a solution of 0.1% toluidine blue/0.1% sodium borate followed by extensive washing with water. Pit formation was imaged using an Olympus dissecting microscope at 1.5x and 4.5x magnification.

### Fluorescence microscopy

For BMV109-labeling of osteoclasts, cells were differentiated in 8-well coverglass chambers (Lab-Tek). BMV109 was added at 1 μM for 2 hours followed by 3 washes in PBS, fixation in 4% paraformaldehyde and DAPI staining. Cells were imaged immediately in PBS using a Zeiss Cell Observer spinning disk confocal microscope with a 20x air objective.

### Osteoclast surface area determination

Osteoclasts were differentiated as described above, except 500,000 cells were seeded in 6-well plates. On day 7, bright field photos were taken of 50 random fields of view with a 10x objective. Surface areas of >250 osteoclasts were then measured using Image J software. Averages were reported with error bars representing standard error of the mean.

## SUPPLEMENTARY FIGURES



## Abbreviations

MDSC: myeloid-derived suppressor cell
RANKL: Receptor activator of nuclear factor kappa-B ligand
M-CSF: macrophage colony stimulating factor
CFSE: carboxyfluorescein succinimide ester
