# Supplementary material for: Cysteine cathepsin activity suppresses osteoclastogenesis of myeloid-derived suppressor cells in breast cancer
Source: Oncotarget. 2015 Jul 17;6(29):27008–22. doi: 10.18632/oncotarget.4714 (PMC4694970; doi:10.18632/oncotarget.4714)
Supplement: Supplementary file 1 [file oncotarget-06-27008-s001.pdf]

SUPPLEMENTARY FIGURES

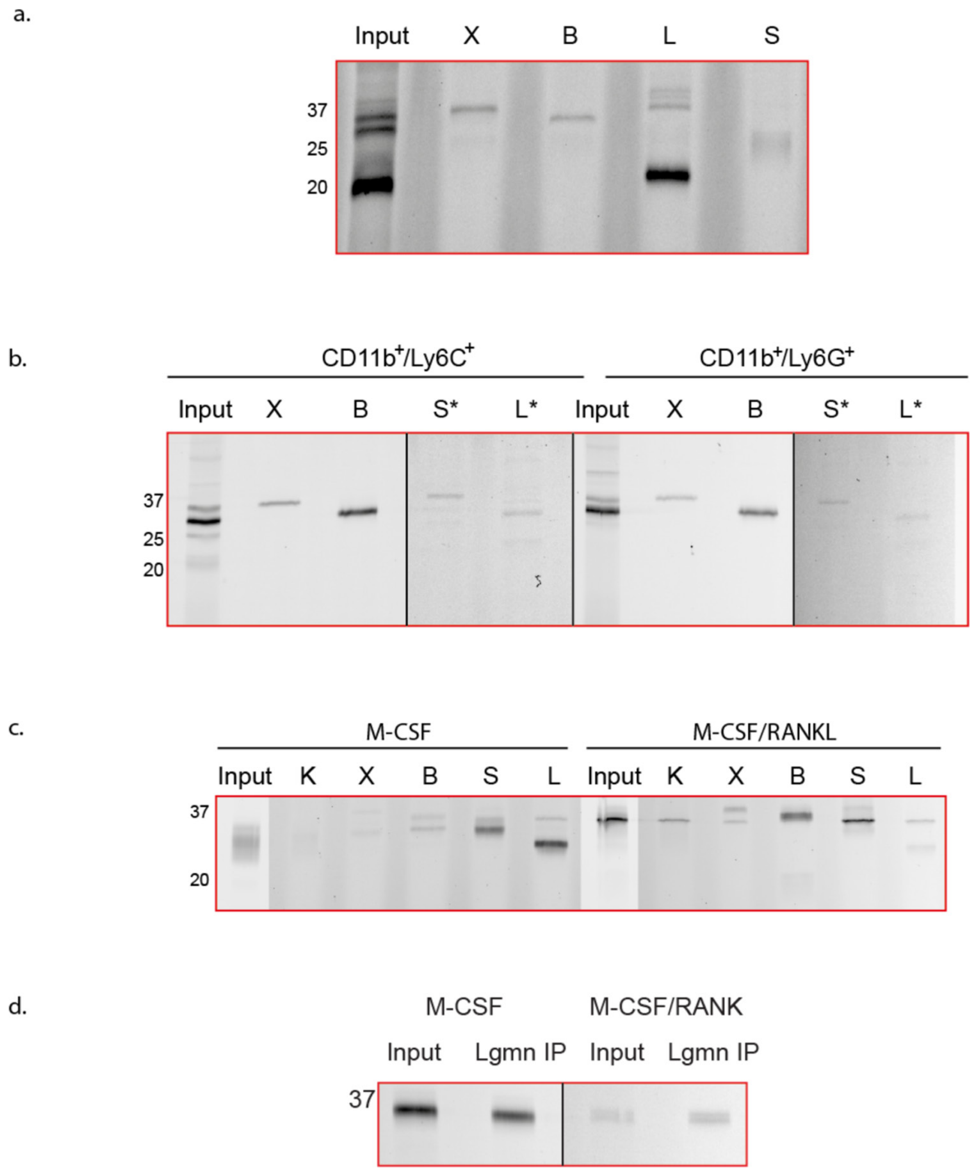

Supplementary Figure S1: Immunoprecipitations confirm the identity of BMV109-labeled proteases. a. Immunoprecipitations of primary tumor samples labeled with BMV109 in Figure 1. **b.** Immunoprecipitations of MDSCs labeled with BMV109 in Figure 2. Contrast of faint bands was increased for ease of viewing, as indicated by an asterisk. **c.** Immunoprecipation of BMV109-labeled macrophage and osteoclast lysates from Figure 5. **d.** Immunoprecipation of LE28-labeled macrophage and osteoclast lysates from Figure 7. Note: this assay is not quantitative given disparities in antibody affinity.

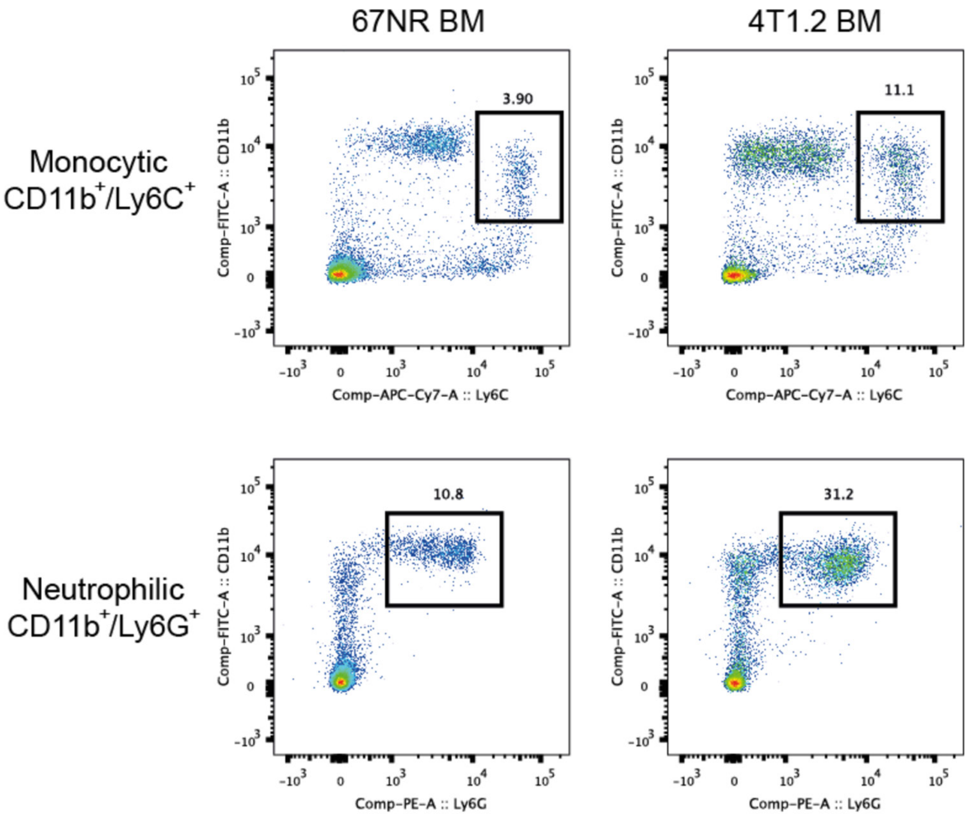

Supplementary Figure S2: MDSC populations are increased in bones bearing metastases. Representative FACS plots corresponding to the graph in Figure 2c. The percentage of $CD11b^+/Ly6C^+$ (monocytic) and $CD11b^+/Ly6G^+$ (neutrophilic) MDSCs in bone marrow is increased in mice bearing 4T1.2 tumors compared to 67NR.

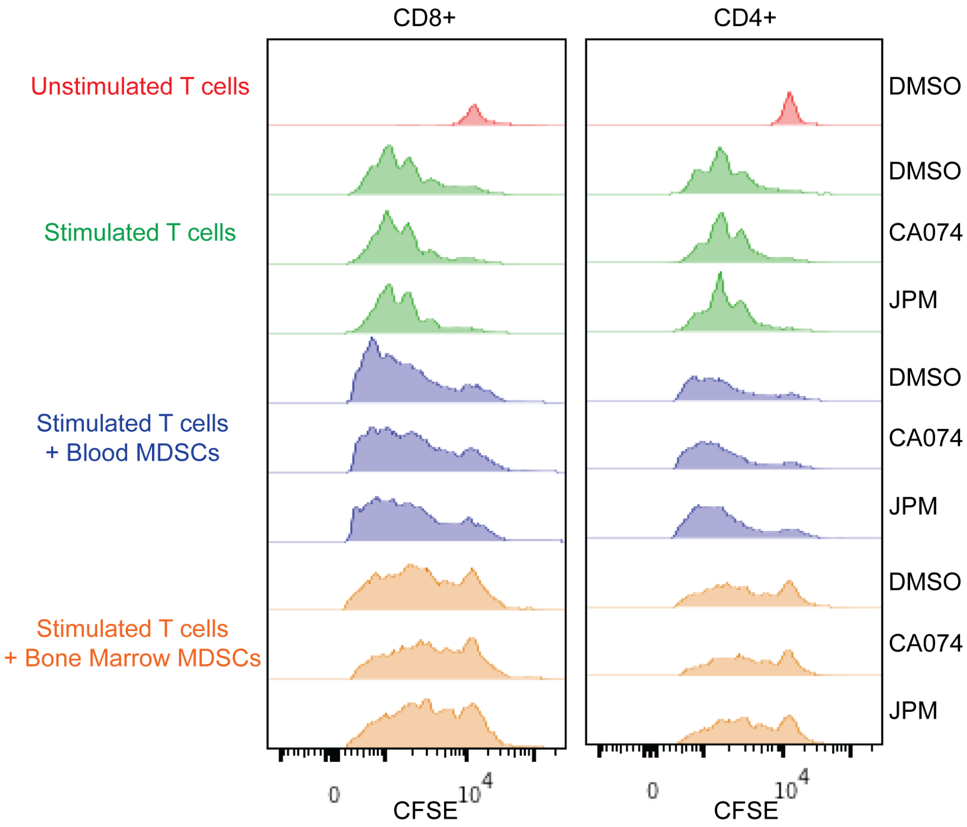

Supplementary Figure S3: Cysteine cathepsin activity is not required for immunosuppressive functions of MDSCs. CFSE-labeled T cells were stimulated to proliferate in the absence and presence of MDSCs purified from blood or bone marrow of mice with experimental 4T1.2 metastasis. A dilution of CFSE (shift to the left) indicates proliferation. Cathepsin inhibitors added at the time of stimulation (CA074 or JPM-OEt) did not affect T cell proliferation or suppression by MDSCs.

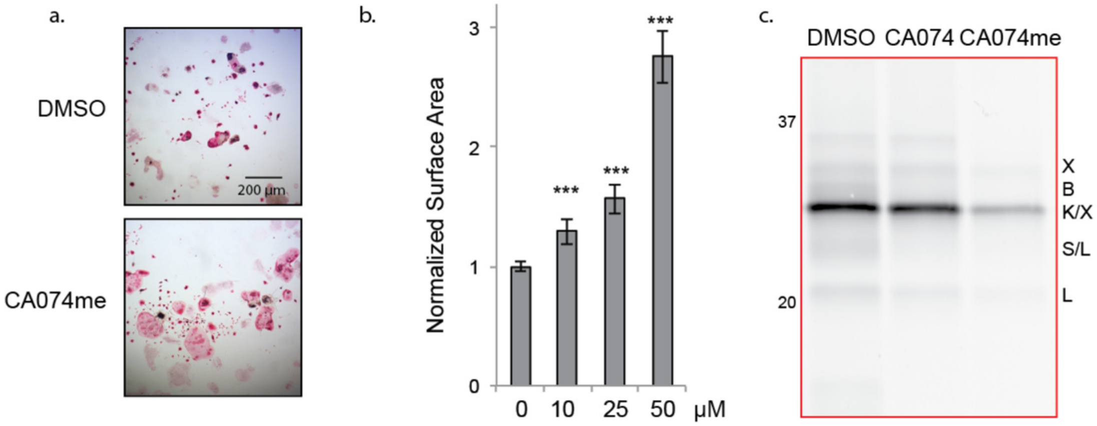

Supplementary Figure S4: CA074me enhances osteoclastogenesis. a. TRAP staining of osteoclasts differentiated with M-CSF/RANKL with DMSO or CA074me. **b.** Average surface area of osteoclasts differentiated with increasing concentrations of CA074me. > 250 cells were measured for each condition. Error bars represent SEM. ***$p < 0.001$ **c.** BMV109 labeling of osteoclasts treated with CA074 and CA074me, indicating that CA074me is not cathepsin-B selective.
